# Prospective analysis of transvalvular systolic flow and aortic wall changes in bicuspid aortic valve insufficiency

**DOI:** 10.1186/1749-8090-8-S1-O21

**Published:** 2013-09-11

**Authors:** E Girdauskas, M Rouman, K Disha, T Scholle, B Fey, B Theis, I Petersen, T Kuntze

**Affiliations:** 1 Department of Cardiac Surgery, Central Hospital Bad Berka, Bad Berka, Germany; 2 Department of Radiology, Central Hospital Bad Berka, Bad Berka, Germany; 3 Department of Pathology, University Hospital Jena, Jena, Germany

## Background

Correlation has been proposed between aortic dilatation patterns and functional status of the bicuspid aortic valve (BAV) (i.e., stenosis vs. insufficiency). The aim of our study was to prospectively evaluate transvalvular systolic flow and proximal aortic lesions in the patients with BAV disease.

## Methods

A total of 76 consecutive patients with BAV disease (57±10 years, 71% male) underwent aortic valve replacement (AVR) ± proximal aortic surgery from January, 2012 through April, 2013. There was a subgroup of 58 (76%) patients with BAV stenosis (Group I), whereas the remaining 18 (14%) patients had isolated BAV insufficiency (Group II). Preoperative cardiac phase-contrast cine-magnetic resonance imaging (MRI) was performed in order to detect the area of maximal flow-induced stress in the proximal aorta. Based on MRI-data, two aortic wall samples i.e., area of the maximal stress (jet-sample) and the opposite aortic wall (control-sample) were collected during surgery. Aortic wall changes were graded based on 7 histological criteria (i.e., each from 0 to 3+) and were summarized in a sum-score. Histological sum-score (0 to 21+) was calculated and compared between samples (i.e., jet- vs. control-sample).

## Results

Eccentric transvalvular jet was identified in all 58 (100%) Group I patients vs. only 5 (28%) in Group II patients (p<0.01). Histological sum-score was significantly higher in jet- vs. control-sample (i.e., 3.9±1.8 vs. 2.1±1.5) (p=0.02) in Group I patients. There was no difference in the histological sum-score between both samples in Group II (i.e., 4.7±3.1 vs. 4.9±3.4) (p=0.7). Histological sum-score was significantly higher in Group II vs. Group I (i.e., 4.9±3.1 vs. 2.3±1.4) (p<0.01).

## Conclusion

Our study confirms the presence of different aortic dilatation patterns in BAV stenosis vs. insufficiency. Our study shows no significant correlation between transvalvular systolic flow pattern and aortic wall lesions in patients with BAV insufficiency.

